# A rare cause of erectile dysfunction: Bilateral thrombosed internal iliac artery aneurysms

**DOI:** 10.12669/pjms.40.8.9005

**Published:** 2024-09

**Authors:** Ibrahim Untan, Ahmed Said Cil

**Affiliations:** 1 Ibrahim Untan Department of Urology, Faculty of Medicine, Ahi Evran University, Kirsehir, Turkiye; 2 Ahmed Said Cil Department of Radiodiagnostics, Faculty of Medicine, Ahi Evran University, Kirsehir, Turkiye

**Keywords:** Erectile dysfunction, Aneurysm, Common iliac artery, Internal iliac artery, Thrombus

## Abstract

Internal iliac artery aneurysms are a relatively uncommon condition that can have serious consequences if left untreated. This paper presents a case of erectile dysfunction caused by thrombosis of the internal iliac artery aneurysms in a 52-years-old male patient with common iliac and internal iliac artery aneurysms. Following the intracavernous self-injection treatment, the patient’s complaints about erectile dysfunction ceased. Aneurysms were monitored closely. To the best of our knowledge, this represents the first case of erectile dysfunction being presented as a consequence of thrombosed iliac artery aneurysms. Erectile dysfunction itself is not a life-threatening condition. However, it should be highlighted that erectile dysfunction can be a sign of life-threatening iliac artery diseases, as in this case. Therefore, it is crucial to recognize the potential seriousness of this condition and to investigate it thoroughly.

## INTRODUCTION

Erectile dysfunction is a prevalent male sexual disorder. It is common in men over the age of 40 affecting more than half of men aged 40-70 years.^1^ A number of different contributing factors are associated with the development of erectile dysfunction. Of these, the most commonly cited are neurogenic, endocrinologic, vasculogenic, and psychogenic factors and medication side effects. Given the high blood flow to the penis, it seems reasonable to posit that the most significant factor in the etiology of erectile dysfunction (ED) is vasculogenic in nature, particularly since the vasculature plays a pivotal role in maintaining penile function. Thus, the decrease in vascular blood flow due to various causes is reflected as erectile dysfunction. The major source of arterial blood flow to the penis is the pudendal artery which is a branch of the internal iliac artery.

Iliac artery aneurysms account for less than 2% of all abdominal aneurysms and affect 0.3% of the general population^2^. Despite the theoretical expectation that iliac artery pathologies would reduce penile blood flow and cause related symptoms, iliac artery aneurysms presenting with erectile dysfunction have not been widely reported in the literature. We present a case of a patient who presented with erectile dysfunction and was diagnosed with bilateral thrombosed internal iliac artery aneurysms following further examination.

## CASE PRESENTATION

**Fig.1(A):**
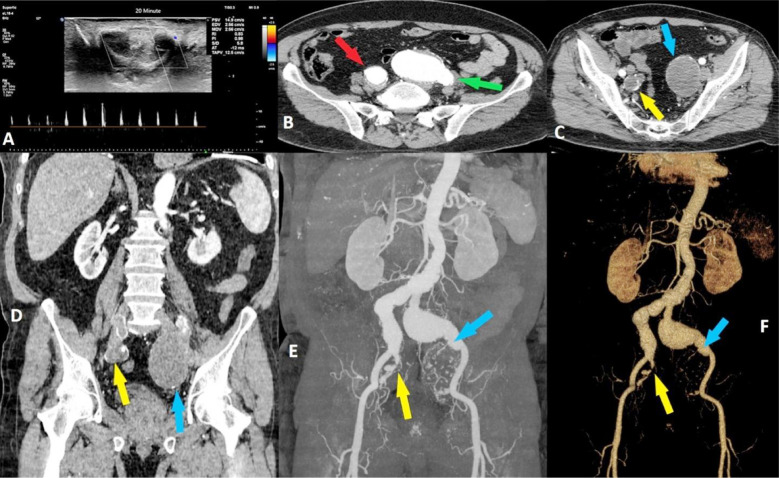
The peak systolic velocity was still around 10-15 cm/sec in post-injection Doppler images obtained at the 20th minute. The cavernous artery was measured 0.4 mm in diameter before injection and no enlargement occurred after the injection. No tumescence and partial rigidity were observed visually. Fig.1(B): Axial computerized tomography section indicating aneurysmatic right and left common iliac arteries with contrast agent included. Fig.1(C): Axial computerized tomography section pointing aneurysmatic right and left internal iliac arteries without contrast agent verifying the complete and almost complete blood flow blockage in the left and right internal iliac arteries respectively. Fig.1(D): Imaging of the explanation in C on the coronal section. Fig.1(E): Aneurysm formations on coronal maximum intensity projection images with almost completely obstructed right internal iliac artery and bo flow in the right internal artery. Fig.1(F): Imaging of the explanation in E in three-dimensional mode. Red arrow: right common iliac artery, green arrow: left common iliac artery, yellow arrow: right internal iliac artery, blue arrow: left internal iliac artery.

A 52-years-old male patient was admitted to the outpatient clinic with a complaint of erectile dysfunction that had persisted for three years. He had undergone coronary bypass surgery for three-vessel coronary artery disease six years ago and has been taking 100 mg of acetylsalicylic acid per day since then. He filled out the International Index of Erectile Function - 5 questionnaire reaching a total score of five points. The patient was 181 cm in height and 87 kg in weight with a calculated body-mass index of 26.5. No notable deviation from normal in the patient’s blood results was observed (Table-I). A 5mg per day tadalafil treatment was planned as initial treatment which was sighted to be failed. Further investigation was conducted via penile Doppler ultrasonography, which revealed evidence of arterial insufficiency, as indicated by a Doppler spectrum flow velocity of approximately 15 cm/s (Fig.1).

**Table-I:** Patient’s blood results.

Parameter (Unit)	Value
Plasma fasting glucose (mg/dL)	101
Creatinine (mg/dL)	0.99
Trygliceride (mg/dL)	196
Total cholesterol (mg/dL)	159
LDL cholesterol (mg/dL)	83
HDL cholesterol (mg/dL)	38
Hemoglobine (g/dL)	15.6
White blood cell (109/L)	6.4
Platelet (109/L)	204

LDL: low-density lipoprotein, HDL: high-density lipoprotein.

Abdominal ultrasonography revealed aneurysms in both the right and left common iliac and internal iliac arteries. Additionally, calcified atheroma plaques and intimal wall thickening were noted in the right and left common iliac artery aneurysms. The Doppler mode, blood flow was completely obstructed in the right internal iliac artery aneurysm and 85% obstructed in the left internal iliac artery aneurysm. Consequently, an aorto-iliac computerized tomography angiography was performed which visualized the aforementioned aneurysms (Table-II). An endovascular aneurysm repair procedure was recommended by a qualified cardiovascular surgeon. However, the patient was reluctant to pursue this course of action due to concerns about the potential for complications, particularly those related to the occurrence of an endoleak. Despite being fully informed about this potential risk, the patient elected to decline the recommended surgical intervention and opted for an alternative course of action. As the underlying vascular problem could not be addressed by other means, urologists proposed a penile prosthetic implant as an alternative solution, but the patient was reluctant to pursue this course of action. After being instructed by the urologist, the patient’s erectile function was maintained by intracavernous self-injection of a mixture containing 15 mg papaverine hydrochloric acid, 1 mg phentolamine mesylate, and 10 µg prostaglandin E1 on demand. The aneurysms were monitored closely by cardiovascular surgeons with the maintenance of current antithrombotic treatment.

**Table-II:** Patient’s aneurysm properties’ details.

Localization	Length (mm)	Diameter (mm)	Thrombose and obstruction
Right common iliac artery	85	35	No thrombose
Left common iliac artery	96	52	No thrombose
Right internal iliac artery	51	29	%85 obstructing thrombose
Left internal iliac artery	76	51	%100 obstructing thrombose

## DISCUSSION

Erectile dysfunction is defined as the situation of inability to achieve and/or maintain penile erection sufficient for satisfactory sexual performance, which is primarily segmented as psychological and organic based on the etiology. Organic erectile dysfunction includes vascular, neurological, endocrinological, and mechanical disorders. Given the robust link between endothelial dysfunction and organic erectile dysfunction, the majority of individuals presenting with the former are typically considered to have a vascular etiology. Nevertheless, erectile dysfunction may develop independently of endothelial dysfunction due to poor arterial flow. In the current case, the patient exhibited symptoms of erectile dysfunction as a consequence of thrombosis of aneurysms that involved the bilateral iliac arteries.

An iliac artery aneurysm is defined as a dilatation of the iliac artery of 1.5-fold the normal diameter. Accordingly, a common iliac artery >18 mm in men and >15 mm in women, and an internal iliac artery >8 mm is considered aneurysmal.^3^ Isolated iliac artery aneurysms are uncommon having a prevalence of less than 2% of all aneurysmal diseases, with 10% internal artery involvement.^4^ One-third of the cases are bilateral.^5^ Iliac artery aneurysms are frequently identified as the result of screening procedures or imaging studies, and in the majority of cases, they do not present with any associated symptoms. However, when aneurysms enlarge sufficiently to exert pressure on neighboring structures or to rupture, symptoms such as abdominal pain may occur.^6^ Rupture is a particularly serious complication, as it can result in life-threatening bleeding.

Furthermore, they can also present with brutal complications such as organ ischemia, vascular-organ fistulas, embolism, intestinal obstruction, or perforation.^7^ Isolated iliac artery aneurysms are divided into four types according to Reber’s isolated iliac artery aneurysms classification, and Type III, which the present case belongs to, accounts for 10% of them (Fig.3).^8^,^9^

In our case; the patient had been smoking for 20 years until having undergone a bypass surgery six years ago. Smoking negatively affects erectile function, and current knowledge most strongly suggests that smoking increases the risk of erectile dysfunction occurrence showing a dose-response correlation with the smoking duration and intensity. In line with the ongoing debate surrounding the restoration of sexual function following smoking cessation, the effects of cessation were not evident in our patient. Hyperlipidemia is one of the risk factors leading to erectile dysfunction, which is a common disorder in men, especially in older men. However, no picture compatible with hyperlipidemia was found in our patient’s blood values. Furthermore, our case did not exhibit any signs of atherosclerosis, as evidenced by the imaging studies presented. Erectile dysfunction is a common complaint prevalent issue among hypertensive men and may be indicative of a systemic vascular disorder, or an adverse effect of antihypertensive medication. Nevertheless, the patient’s hypertension did not necessitate the administration of antihypertensive agents. One-quarter of cases of erectile dysfunction are attributable to drug therapy. Nonetheless, in our case, the only medication was acetylsalicylate 100 mg a day, which made it unlikely that drug-related erectile dysfunction was the cause. In the current case, erectile dysfunction due to arterial insufficiency evinced thrombosis of aneurysms involving the bilateral iliac arteries. Although cases presenting with hydronephrosis and flank pain as urological manifestations have been reported, to our knowledge, this is the first case of an aneurysm case presenting with erectile dysfunction in the literature.^10^ Clinicians should not always attribute erectile dysfunction to advancing age or mental status but should keep in mind that there may be life-threatening underlying causes such as aneurysm or thrombosis as in the present case.

### Availability of data and materials

The data that support the findings of this study are available upon reasonable request from the corresponding author.

### Ethical approval

All procedures performed in the studies involving human participants were per the ethical standards of the institutional and/or national research committee and with the 1964 Helsinki Declaration and its later amendments or comparable ethical standards. For this type of study formal consent is not required.

### Informed consent

Informed consent was obtained from the patient at every treatment step throughout the entire treatment process in which a healthcare professional educated the patient about the risks, benefits, and alternatives of a given procedure or intervention. In the form, the patient has given his consent for clinical information to be reported in the journal. The patient understands that their names and initials will not be published and due efforts will be made to conceal their identity, but anonymity cannot be guaranteed.

### Authors’ Contributions:

**IU and ASC:** Conceived and designed the study.

**ASC:** Collected data.

**IU:** Reviewed the literature and prepared the manuscript.

**IU and ASC:** Provided critical feedback to each other and contributed to the final manuscript after discussing the results and commenting on the manuscript, read and approved the revised version of the manuscript, and ratified the order of authors.
